# Guar-Based Injectable Hydrogel for Drug Delivery and In Vitro Bone Cell Growth

**DOI:** 10.3390/bioengineering10091088

**Published:** 2023-09-15

**Authors:** Humendra Poudel, Ambar B. RanguMagar, Pooja Singh, Adeolu Oluremi, Nawab Ali, Fumiya Watanabe, Joseph Batta-Mpouma, Jin Woo Kim, Ahona Ghosh, Anindya Ghosh

**Affiliations:** 1 Department of Chemistry, University of Arkansas at Little Rock, 2801 South University Avenue, Little Rock, AR 72204, USA; hxpoudel@ualr.edu (H.P.); ahonag7@gmail.com (A.G.); 2 Department of Chemistry, Philander Smith University, 900 W Daisy L Gatson Bates Dr, Little Rock, AR 72202, USA; arangumagar@philander.edu; 3 Department of Biology, University of Arkansas at Little Rock, 2801 South University Avenue, Little Rock, AR 72204, USA; pxsingh1@ualr.edu (P.S.); asoluremi@ualr.edu (A.O.); nali@ualr.edu (N.A.); 4 Center for Integrative Nanotechnology Sciences, University of Arkansas at Little Rock, 2801 South University Avenue, Little Rock, AR 72204, USA; fxwatanabe@ualr.edu; 5 Department of Biological and Agricultural Engineering, Bell Engineering Center, University of Arkansas, 4183 Fayetteville, Little Rock, AR 72701, USA; jnbattam@uark.edu (J.B.-M.); jwkim@uark.edu (J.W.K.)

**Keywords:** modified natural polymer, injectable hydrogel, drug delivery, drug release kinetics, bone tissue engineering

## Abstract

Injectable hydrogels offer numerous advantages in various areas, which include tissue engineering and drug delivery because of their unique properties such as tunability, excellent carrier properties, and biocompatibility. These hydrogels can be administered with minimal invasiveness. In this study, we synthesized an injectable hydrogel by rehydrating lyophilized mixtures of guar adamantane (Guar-ADI) and poly-β-cyclodextrin (p-βCD) in a solution of phosphate-buffered saline (PBS) maintained at pH 7.4. The hydrogel was formed via host-guest interaction between modified guar (Guar-ADI), obtained by reacting guar gum with 1-adamantyl isocyanate (ADI) and p-βCD. Comprehensive characterization of all synthesized materials, including the hydrogel, was performed using nuclear magnetic resonance (NMR) spectroscopy, Fourier transform infrared (FTIR) spectroscopy, scanning electron microscopy (SEM), energy dispersive X-ray spectroscopy (EDS), X-ray diffraction (XRD), thermogravimetric analysis (TGA), and rheology. The in vitro drug release study demonstrated the hydrogel’s efficacy in controlled drug delivery, exemplified by the release of bovine serum albumin (BSA) and anastrozole, both of which followed first-order kinetics. Furthermore, the hydrogel displayed excellent biocompatibility and served as an ideal scaffold for promoting the growth of mouse osteoblastic MC3T3 cells as evidenced by the in vitro biocompatibility study.

## 1. Introduction

Hydrogels are excellent materials for diverse applications including tissue engineering, and drug delivery [1]. Hydrogels’ unique three-dimensional (3D) porous structure, which retains a large amount of water, flexibility, biocompatibility, and biodegradability, furnishes the hydrogels as excellent candidates for myriad biomedical uses [2]. Polymeric injectable hydrogels have emerged as captivating subjects of research in the biomedical field owing to their distinctive properties, including tunability, mechanical flexibility, and most notably, their ability to be injected with low invasiveness for patient implantation [3,4,5,6]. Most injectable hydrogels have been developed, as described in the literature, by employing external stimuli such as temperature via physical crosslinking methods [7,8,9,10]. Injectable hydrogels can be developed using different types of polymers such as natural and synthetic polymers in different ways and are employed in the biomedical field [11,12]. It has been reported that temperature-sensitive poly(N-isopropylacrylamide) hydrogel was successfully prepared and its applications for bone tissue engineering and drug delivery were studied in detail [13]. The injectable hydrogel can also be prepared based on the pH [14]. Besides temperature and pH, light, heat, electricity, and magnetic fields have been used to develop injectable hydrogels [15].

The poor stability and low mechanical properties are the main issues associated with physical crosslinking methods [16]. Host-guest chemistry can be used to circumvent such limitations even though host-guest chemistry belongs to the physical cross-linking method [17]. Host-guest chemistry has also been exploited to develop physically crosslinked hydrogels for different biomedical applications [18,19,20]. The host can interact with polymers or small guest molecules that can fit in the host’s cavity, thereby crosslinking them to form gels [21]. Cyclodextrin (CD), crown ether (CE), and cucurbituril (CB) are the most commonly used hosts [22]. The inner hydrophobic cavity of CD can interact with polymers such as polyethyleneglycol (PEG) or polypropylene glycol (PPG) and small molecules such as ferrocene, adamantine (AD), and azobenzene which can lead to crosslinking process [23]. Similarly, CB has been used to gel naphthalene-functionalized hydrophilic polymers [24]. Such hydrogels exhibited intriguing mechanical properties and have been applied in the development of drug delivery systems and tissue engineering for stem and bone cells [25,26]. Although progress has been made to develop physically crosslinked hydrogel using host-guest chemistry, economic approaches to establish a stable biodegradable and biocompatible hydrogel using natural polymers are rare in the literature and thus provide motivation for further study.

**Figure 1:**
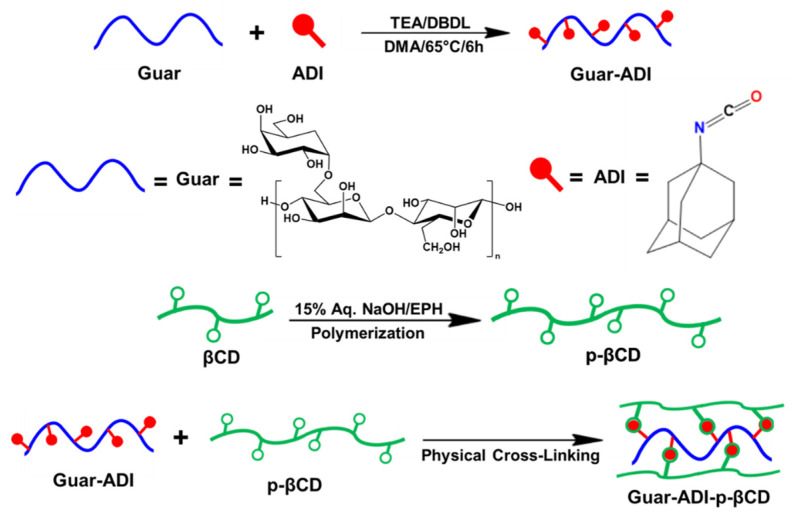
The schematic diagram for the synthesis of Guar-ADI, p-βCD, and Guar-ADI-p-βCD hydrogels.

In this manuscript, we present the synthesis of an injectable hydrogel using guar gum as the main polymeric material. Guar gum, a cost-effective, biodegradable, and biocompatible polymer, is suitable for applications such as drug delivery and tissue engineering [27]. As guar gum contains several hydroxyl groups, we modified it with 1-adamantyl isocyanate (ADI) to synthesize Guar-ADI [28]. We used β-cyclodextrin (βCD) polymer for this research to make the injectable hydrogel [29]. βCD is poorly soluble in water hence it was polymerized to poly-β-cyclodextrin (p-βCD) with epichlorohydrin (EPH) to make it water soluble [30,31,32]. This report illustrates the synthesis of a hydrogel formation via host-guest interaction using Guar-ADI solution with an aqueous solution of p-βCD (Figure 1). Furthermore, we report the investigation of the injectable hydrogel for in vitro BSA and anastrozole release and conduct an in vitro biocompatibility study by checking the growth of mouse osteoblastic MC3T3 cells. These findings contribute to the advancement of hydrogel-based drug delivery systems and tissue engineering.

## 2. Materials and Methods

### 2.1. Materials

The reagents and solvents used in this study were sourced from reliable commercial suppliers and employed without further purification unless otherwise stated. Guar Gum was procured from VWR BeanTown Chemical (Hudson, NH, USA). ADI was purchased from Acros Organics (Pittsburgh, PA, USA). BSA (Cohn fraction, molecular weight 66 kDa) and phosphate buffered saline (10X, pH 7.4) were acquired from VWR Life Science (Radnor, PA, USA). Anastrozole was purchased from Bio-Vision (San Franscisco, CA, USA). Hydrochloric acid (HCl), sodium hydroxide (NaOH), and deionized water were obtained from VWR BDH Chemicals (Rouses Point, NY, USA). βCD was procured from TCI America (Portland, OR, USA). Dimethylsulfoxide-D6, ethanol, and isopropanol were sourced from Millipore Sigma (Burligton, MA, USA). Triethylamine (TEA), dibutyltin dilaurate (DBDL), epichlorohydrin (EPH), toluene, N,N-dimethylacetamide (DMA), and cell counting kit-8 were procured from Sigma-Aldrich (St. Louis, MO, USA). Minimum Essential Medium-Earle’s (MEM) and fetal bovine serum (FBS) were acquired from Caisson Labs (Smithfield, UT, USA).

### 2.2. Synthesis of Guar-ADI Compound

The synthesis of Guar-ADI was carried out using a modified version of the literature method [33]. Initially, 2 g (g) of guar gum was taken in a three-necked round bottom flask (RBF). Subsequently, 25 mL of DMA was added to the RBF. To this solution, 1 equivalent of ADI (1.947 g, 10.99 mmol) was introduced while maintaining a constant stirring at 65 °C using an oil bath. After 30 min of stirring, 0.15 mL of TEA (1.076 mmol) and 0.65 mL of DBDL (1.092 mmol) were sequentially added and stirred for an additional 6 h. Afterward, the resulting mixture underwent filtration and sequential washing with hexane, acetone, and isopropanol, each performed at least three times. Finally, the product was dried in a vacuum oven, yielding a powdered final product. A total of 1.986 g of the product was obtained.

### 2.3. Polymerization of β-Cyclodextrin

The polymerization of βCD was conducted following a literature method [34]. Initially, 10 g of βCD (8.810 mmol) were dissolved in 10 mL of a 15% aqueous NaOH solution, after which 10 mL of toluene was added. The mixture was stirred for approximately 2 h at 35 °C. Subsequently, 5 mol% of EPH (3.92 mL, 50 mmol) was introduced and stirred for an additional 3 h at a temperature of 55 °C. To neutralize the reaction mixture, 6 M HCl solution was added dropwise to maintain pH 7 of the solution. The resulting product was precipitated by adding copious amounts of isopropanol. The precipitate was collected through filtration, and the product was then dried in a vacuum oven to obtain it in powder form. The mass of the final product obtained was 8.2 g.

### 2.4. Preparation of Hydrogel

The different concentrations of hydrogel were prepared by mixing rehydrated equal volumes of different concentrations of Guar-ADI and p-βCD solutions prepared in PBS (pH 7.4) under ambient temperature. The hydrogel material was mixed with the required concentrations of BSA and anastrozole prepared in PBS solution separately.

### 2.5. Characterizations of Materials

The guar gum, guar-ADI, βCD, p-βCD, and hydrogels were characterized by different spectroscopic and microscopic techniques such as FTIR Spectroscopy, NMR Spectroscopy, TGA, SEM, EDS, XRD, and Rheology. UV-visible spectroscopy was used for the determination of concentrations of drugs released from the hydrogel.

The FTIR spectroscopy of the sample was conducted by using a Nicolet 6700 Thermo-Scientific FTIR spectrometer. The FTIR spectra were recorded from 400 to 4000 cm^−1^. The NMR spectroscopy was performed using a JEOL ECS-type nuclear magnetic resonance spectrometer with a frequency of 400 MHz. The TGA technique was used to study the weight loss of Guar gum, and guar-ADI compound at various temperatures. The TGA analysis was successfully recorded using a Mettler Toledo thermogravimetric analyzer DSC3+. The dried hydrogel’s surface morphology was examined using a JEOL scanning electron microscope (JSM 7000F) equipped with an EDS system. SEM images were captured at different magnifications to investigate the hydrogel’s surface morphology. The EDS investigation of the dried hydrogel was achieved using the JEOL scanning electron microscope (JSM 7000F) with the EDS system. XRD patterns of the dried hydrogels and their components were measured using XRD analysis. The XRD analysis of the hydrogel was conducted using the Bruker D8 Discover instrument. Rheological characterization of all hydrogel samples was conducted using a dynamic hybrid rheometer (DHR)-2 from TA Instruments (New Castle, DE, USA) with a 40 mm diameter flow ramp and a 1° angle cone plate geometry. Oscillation (frequency) sweep experiments were studied at 25 °C to determine the viscoelastic response of the gel. Flow ramp experiments were conducted at a 1% strain and a frequency of 1 Hz to evaluate the flow behavior and shear thinning properties with varying levels of viscosity based on gel concentration. Oscillation frequency sweep experiments were executed at an angular frequency ranging from 10 to 1000 rad/s and a 1% strain to assess the mechanical response under deformation. The concentrations of drugs (BSA and anastrozole) were determined using a Perkin Elmer Inc. double-beam UV-Visible spectrophotometer operating in the 200 to 700 nm range.

### 2.6. Measurement of the Swelling Property

To study the hydrogel’s swelling behavior, excess water was removed by wiping the hydrogel with tissue paper, and the initial weight was recorded. Subsequently, the hydrogel was immersed in PBS buffer at pH 7.4 for a duration of 24 h. After carefully blotting away the excess buffer with tissue paper, the swollen hydrogel’s final weight was recorded. The swelling percentage (%) of the hydrogel was calculated using the following formula:

$$Swelling\;\%=\left[\frac{W_{t}-W_{o}}{W_{o}}\right]\times 100$$

Here, W_t_ is the weight of the hydrogel at time t, and W_o_ represents the hydrogel’s initial weight.

### 2.7. In Vitro Drug Release Study

To study in vitro drug release, BSA was employed as a hydrophilic drug, while anastrozole served as a hydrophobic drug, as indicated in the literature [35]. The study was conducted using a 5% hydrogel at a temperature of 37 °C in PBS. To initiate the experiment, 5% (*w*/*w*) hydrogels with varying concentrations of BSA (1, 3, and 6 mg/mL) and anastrozole (50, 250, and 500 μg/mL) were prepared. This was achieved by rehydrating 100 mg of lyophilized mixtures in PBS (pH 7.4) at a 50/50 ratio (*w*/*w*). During the experiment, a 3 mL aliquot was taken out at specific time intervals to assess the drugs released from the hydrogel. To maintain sink conditions, a fresh media of 3 mL was replenished after each withdrawal. The concentrations of the released drugs were analyzed and calculated based on the wavelength maxima for BSA at 280 nm and anastrozole at 263 nm using UV-vis spectroscopy.

### 2.8. Cell Culture

All cell culture and biocompatibility studies performed are ethically in compliance. They were conducted in a Biosafety level-2 (BSL-2) facility approved by the University of Arkansas at Little Rock’s Institutional Biosaftey Committee (protocol approval #00057, 25 April 2012). Mouse osteoblastic cells (MC3T3-E1) were purchased from the American Type Culture Collection (ATCC). The cells were cultured in MEM-Earle’s medium supplemented with 10% FBS and penicillin (500 units/mL), and streptomycin (500 units/mL) as described earlier [36]. The cell culture was maintained in a controlled humidified environment at 37 °C with a 5% CO_2_ atmosphere in an incubator and subcultured at approximately 80% confluence. For subculture, cells were washed with PBS solution at pH 7.4 and detached with 0.05% Trypsin/0.53 mM EDTA solution. Trypsin/EDTA solution was removed by centrifugation. Live cells were counted by hemocytometer following trypan blue staining before plating on scaffold materials for biocompatibility assessment [36]. The medium was refreshed every 2–3 days to ensure optimal cell growth and viability.

### 2.9. Biocompatibility Assay

The biocompatibility assessment of the hydrogel was conducted using Mouse osteoblastic cells (MC3T3-E1). These cells serve as a widely accepted model for testing any application for bone implants. Cell viability was determined using a cell counting kit-8 (CCK-8) assay, which is a method used to determine viable cell counts, reflecting cell growth and viability. In this assay, control samples, hydrogel scaffolds, and hydrogel scaffolds containing test drugs were added to 96-well plates under sterile conditions and allowed to settle overnight to form a thin film substratum before seeding the cells on them. Approximately 5 × 10^3^ MC3T3-E1 cells per well were seeded onto the plates and cultured in a humidified incubator maintained at 37 °C with 5% CO_2_ for 24 h, 48 h, and 72 h to allow for cell growth and interaction with the hydrogel. After the designated incubation period, 10 µL of the cell counting kit-8 assay solution was added to every well of the plate. The plate was then further incubated at 37 °C for 4 h more. Subsequently, the absorbance of the samples was measured using a microplate reader at 450 nm (Synergy H4, Bio-Tek). This measurement allowed for the quantification of cell viability, which is indicative of the cell growth on the hydrogel samples. The percentage of cell viability was calculated as follows.

Cell viability (%) = [(A_s_ − A_b_)/(A_c_ − A_b_)] × 100,

where,

A_s_ = Experimental well’s absorbance (absorbance of cells, medium, CCK8 and wells of the hydrogel materials).

A_b_ = Absorbance of the blank well (absorbance of wells containing medium and CCK8).

A_c_ = Absorbance of the control well (absorbance of wells containing cells, medium and CCK8).

### 2.10. Statistical Analysis

The results for biocompatibility studies are expressed as a mean ± SD from at least 3 independent experiments each conducted in quadruplicate wells. We used a one-way ANOVA test to evaluate the statistical significance between the control and experimental groups. The *p*-value of *p* ≤ 0.05 was considered as the data with significant difference compared to controls.

## 3. Results and Discussions

### 3.1. Characterizations

The modified guar with 1-adamantyl isocyanate and polymerized βCD samples were subjected to FTIR spectroscopy analysis for characterization. Figure S1 displays (Supporting Information) the FTIR spectra of Guar, ADI, and modified guar (guar-ADI). In the Guar-ADI spectrum, a broad peak ranging from 3584 to 3700 cm^−1^ indicates the combined O-H and N-H stretching. The sharp peaks observed at 2850 cm^−1^ to 2930 cm^−1^ correspond to -CH stretching, which is present in all compounds (guar, ADI, and Guar-ADI) [37]. Furthermore, the peak at 2250 cm^−1^ corresponds to the N=C=O group of ADI [38]. The peak at 1620 cm^−1^ indicates the existence of the C=O functional group associated with the urethane compound found in Guar-ADI, thus confirming the modification of guar gum with ADI. Figure S2 (Supporting Information) indicates the FTIR spectra of βCD and p-βCD. The FTIR spectrum of βCD shows a broad peak at 3300 cm^−1^ which is due to hydroxyl (-OH) stretching, whereas the intense peak at 2920 cm^−1^ is due to C-H (symmetric and asymmetric stretching vibrations) bonds in the molecule. The peak at 1640 cm^−1^ is due to the H-OH deformation peak of the adsorbed water in βCD [39]. The 1150 cm^−1^ and 1020 cm^−1^ peaks show the peaks of C-H overtone and C-O stretching frequency, respectively. The peak at 1150 cm^−1^ also shows the peak of C-O-C vibration. All the peaks matched the literature-reported data [39]. After the polymerization of βCD, the peaks were slightly shifted, and no new peaks were observed because p-βCD and βCD both contain similar functional groups. The shift observed from 2920 cm^−1^ to 2930 cm^−1^ is attributed to the overlapping of C-H peaks of EPH with βCD.

The modification of guar gum with ADI and the polymerization of βCD to p-βCD was further characterized using the ^1^H-NMR spectroscopy technique. Figure S3 (Supporting Information) depicts the ^1^H-NMR of Guar-ADI in D_2_O solvent. The NMR peaks between 3–4 ppm are due to the peaks of guar gum, which agree with the literature-reported peaks [40]. Figure S4 (Supporting Information) presents the ^1^H-NMR spectra of p-βCD in deuterium oxide (D_2_O) solvent. The ^1^H-NMR of p-βCD shows six different peaks from 1.1–5.0 ppm. The two extra peaks at 3.7 and 3.8 ppm are present only in p-βCD but not in βCD, confirming the compound to be p-βCD [41]. Figure S5 (Supporting Information) shows ^13^C NMR of Guar-ADI, which shows peaks in the range of 6–174 ppm. The peaks at 63, 70, and 100 ppm are due to guar gum, and the remaining peaks are due to ADI and EPH moieties [42]. The number of carbon peaks confirms that guar was modified with ADI.

**Figure 2:**
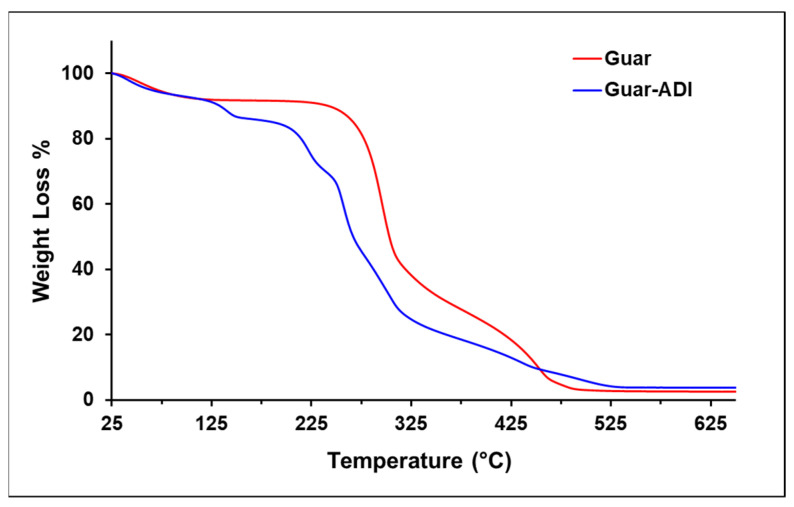
TGA plot of Guar and Guar-ADI.

The modification of Guar gum with ADI was investigated using the TGA technique, as depicted in Figure 2, which illustrates the recorded weight loss of the samples at different temperature ranges. Initially, both guar gum and guar-ADI exhibited weight loss between 25 °C and 258 °C, accounting for approximately 10% due to moisture loss [43]. The weight loss was more pronounced in Guar-ADI compared to Guar gum, indicating a higher moisture content in the Guar gum upon modification with ADI. Another weight loss occurred between 225 °C and 320 °C, corresponding to approximately 65% weight loss attributed to the loss of hemiacetal [44]. Subsequently, at 481 °C, guar gum experienced a significant 95% weight loss because of the decomposition of the polymer backbone, hemiacetal, and moisture loss [45]. In the case of Guar-ADI, a similar mass loss was observed at 530 °C, indicating enhanced thermal stability resulting from the modification with ADI.

The XRD analysis of the dried hydrogel revealed a range of intense peak 2ϴ values spanning from 15° to 84°. Notably, Figure S6 (Supporting Information) highlights two prominent peaks at 32° and 45°. In contrast, the X-ray diffraction pattern of guar gum displayed a broad peak at 2ϴ = 18°, indicating its non-crystalline nature due to the small crystallite size (5.05 Å), in accordance with the literature [46]. However, following the synthesis of the hydrogel, the XRD pattern exhibited intense peaks at 2ϴ values of 15°, 27°, 32°, 45°, 54°, 57°, 66°, 75°, and 84°, indicating the presence of crystallinity within the hydrogel. Notably, the peaks at 15° and 27° are characteristic of βCD [47].

**Figure 3:**
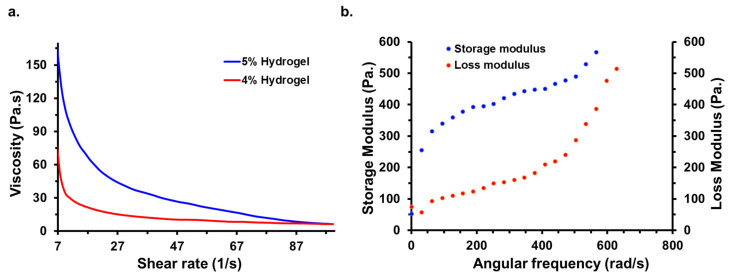
(**a**) Dependence of viscosity on the shear rate of 4% and 5% hydrogels. (**b**) Storage and loss modulus vs. angular frequency of the 4% and 5% hydrogel.

The hydrogel underwent additional characterization through rheology measurements. Figure 3a illustrates the viscosity versus shear rate profiles of 4% and 5% hydrogels. Viscosity is a measure of a hydrogel’s resistance to flow [46]. In this instance, the viscosity exhibits shear-thinning behavior, as viscosity decreases with the increase in the shear rate. This property implies that the hydrogel can be easily injected under shear stress and rapidly recover its structure upon stress removal [48]. Notably, the viscosity of the 5% hydrogel is higher than that of the 4% hydrogel due to the greater polymer concentration, highlighting the strength of the 5% hydrogel. Furthermore, the viscoelastic behavior of the 5% hydrogel was assessed by examining the dynamic moduli as a function of angular frequency, as shown in Figure 3b. Dynamic moduli represent the viscoelastic properties of the hydrogel, representing the ratio of shear stress to shear strain under vibratory conditions. In this analysis, the storage moduli values consistently exceeded the loss moduli values, indicating the predominance of elasticity over viscosity in the entangled network of the hydrogel [48].

Figure S7 (Supporting Information) presents the relationship between the tan delta values of the 4% and 5% hydrogels and the angular frequency. Tan delta is the ratio of the storage modulus to the loss modulus [49]. The storage modulus provides insights into the elastic nature of the hydrogel, while the loss modulus describes its viscous behavior [50]. As the tan delta values for these hydrogels are below 1, it indicates that the hydrogels exhibit a predominantly elastic rather than viscous behavior. Comparing the different hydrogels, the 5% hydrogel demonstrates greater strength, as evidenced by lower tan delta values compared to the 4% hydrogel across all angular frequencies. This suggests a higher degree of entanglement in the 5% hydrogels compared to the 4% hydrogel.

The UV-visible spectroscopy analysis of the hydrogel revealed that the percent transmittance is relatively consistent in both hydrogel samples. For the 4% hydrogel, the percent transmittance was approximately 10, and this value increased with higher concentrations of the hydrogel. In contrast, the 5% hydrogel exhibited a percent transmittance of approximately 19, indicating a higher polymeric concentration in the hydrogel. This higher concentration contributes to the stronger nature of the 5% hydrogel, which restricts the passage of light. Importantly, the gradual change in transmittance suggests that the hydrogel does not exhibit significant absorbance, which is advantageous for conducting drug release studies without interference. Figure S8 (Supporting Information) illustrates the UV-visible spectra of both the 4% and 5% hydrogel samples.

Figure S9 (Supporting Information) depicts the EDS analysis of 5% dried hydrogel to determine its elemental composition. The EDS analysis of the 5% hydrogel revealed the relative abundance of constituent elements. By weight, the hydrogel comprises approximately 52.7% carbon, 30% nitrogen, and 17.4% oxygen. These data confirm the presence of carbon, nitrogen, and oxygen in the hydrogel without any detectable impurities, further supporting its suitability for biomedical applications.

For the swelling study, 0.558 g of a 5% hydrogel sample was utilized. This hydrogel was immersed in deionized distilled water for 24 h, and its mass was measured. Following the swelling process in distilled water, the mass of the hydrogel was found to be 3.22 g. Therefore, the swelling percentage of the hydrogel was determined to be 477%. The swelling study of the 5% hydrogel demonstrated its impressive capacity to absorb water, approximately five times its weight or 477 times its original volume. This feature makes the hydrogel highly valuable for various biomedical applications.

### 3.2. In Vitro Drug Release Study

The investigation of controlled drug release using an injectable hydrogel represents a significant area of research, as mentioned earlier. In our study, we specifically examined the hydrogel’s capability for controlled drug release under in vitro conditions, focusing on two different substances: BSA and anastrozole. BSA was chosen as a model protein for hydrophilic drugs due to its prevalence as the third-most abundant whey protein in milk. Comprising a single chain of 583 amino acid residues, BSA has a molecular mass of 66.5 kDa [51]. It is one of the major proteins in biological fluids and is commonly utilized as a protein supplement in mammal cell culture media [52]. Previous research conducted by Crow et al. explored in vitro BSA release from poly (L-lactic acid)-based hydrogel materials [53], while Nadam et al. investigated the BSA release kinetics using a hydrogel of poly(N-isopropylacrylamide) [54]. Furthermore, we examined anastrozole, an anticancer drug, as a representative hydrophobic drug for in vitro release studies [55]. Anastrozole is a non-steroidal aromatase inhibitor primarily used to treat postmenopausal women with estrogen-responsive breast cancer by reducing their estrogen levels [56]. Tuna et al. studied the kinetics of anastrozole release from silk fibroin rods [57]. However, it is noteworthy that no previous studies have investigated the release of BSA or anastrozole using the Guar-ADI-p-βCD hydrogel, making our approach unique and innovative in the investigation of the hydrogel’s ability in its drug delivery application.

**Figure 4:**
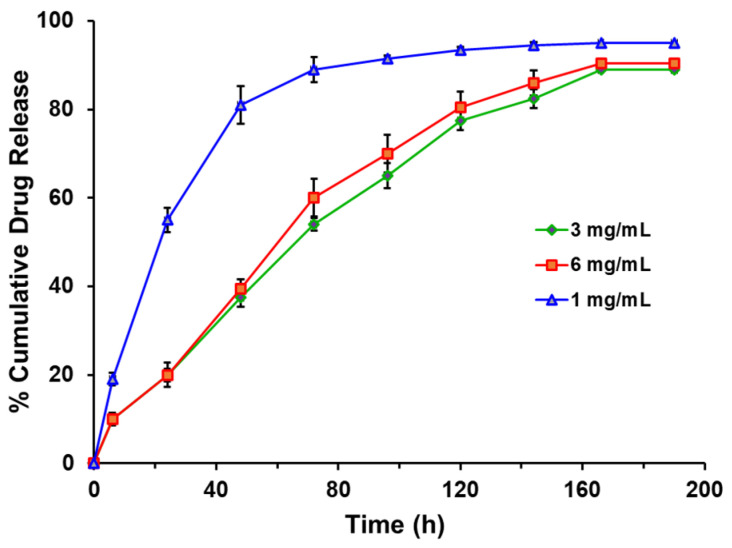
In Vitro BSA release study from 5% hydrogel having 1 mg/mL, 3 mg/mL, 6 mg/mL BSA concentration in PBS (pH 7.4) at 37 °C.

An in vitro BSA release study was conducted using a 5% hydrogel with varying concentrations of BSA (1, 3, and 6 mg/mL) in PBS (37 °C, pH 7.4) for approximately 190 h, as depicted in Figure 4. During the initial 24 h, approximately 40% of the BSA was released from the hydrogel containing 1 mg/mL BSA. In contrast, hydrogels with 3 mg/mL and 6 mg/mL of BSA exhibited less than 20% release. As the BSA concentration increased, the cumulative percentage of drug release decreased. This observation can be attributed to the stronger interaction between the protein and the hydrogel at higher concentrations [35]. The faster release observed at 1 mg/mL loading can be attributed to the relatively low interaction between the proteins and the hydrogel. However, at higher concentrations of drug loading, the protein tends to interact more strongly with the hydrogel, possibly via hydrogen bond formation. This stronger interaction slows down the protein release from the hydrogel matrix.

We then investigated the kinetics of drug release using various models. Firstly, we examined the zero-order kinetics by plotting the graph of the percentage of cumulative drug release against time. However, no linear relationship was observed (Figure 4), indicating that BSA release did not follow zero-order kinetics.

Subsequently, we explored the Higuchi model for drug release. The Higuchi model equation is represented as $Q=K_{H}\;t^{\frac{1}{2}}$, where Q indicates the cumulative amount of drug release at the time ‘t’, K_H_ is the Higuchi constant, and t is the time in hours [58]. Upon plotting the percentage of cumulative release of the drug against the square root of time, we once again observed a lack of linearity in the plot, indicating that BSA release did not follow the Higuchi model (Figure S10a, Supporting Information). To investigate the BSA release study from the 5% hydrogel, we also examined the Weibull Model by plotting Ln[−ln(1 − %Drug release)/100] vs. lnt. However, no linear relationship was observed (Figure S10b, Supporting Information), indicating that the BSA release from the hydrogel did not follow the Weibull Model.

**Figure 5:**
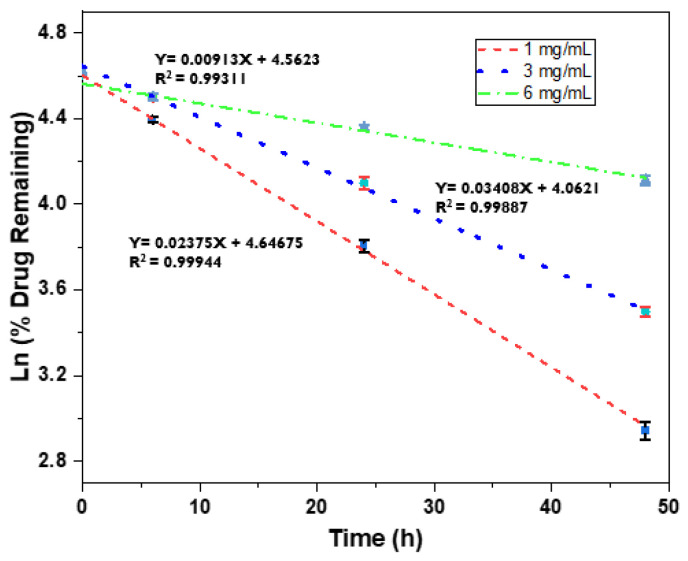
First-order kinetics of BSA release study from 5% hydrogel.

Finally, we investigated the first-order kinetics of BSA released from the 5% hydrogel by plotting the natural logarithm of the percentage of the remaining drug against time, as illustrated in Figure 5. Notably, straight lines were obtained, indicating that BSA was released from the hydrogel material in accordance with first-order kinetics. This finding indicates that the rate of BSA release from the hydrogel is dependent on the concentration of BSA [59].

Rate of release of BSA = K [Conc. of BSA](1)

**Figure 6:**
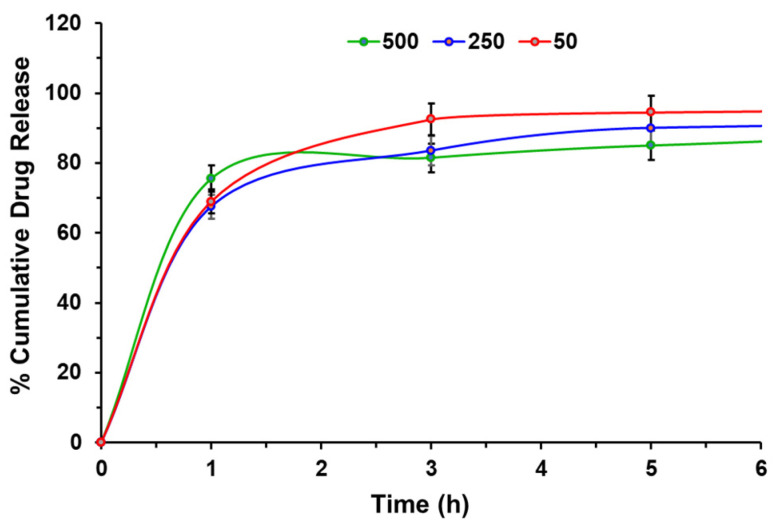
In Vitro drug release study from 5% hydrogel having 50 μg/mL, 250 μg/mL, and 500 μg/mL anastrozole concentration in PBS, pH 7.4.

Additionally, an in vitro study was performed to investigate the release of anastrozole from the 5% hydrogel at varying concentrations (50, 250, and 500 μg/mL) over a duration of 10 h at 37 °C, as depicted in Figure 6. It was observed that approximately 68–75% of anastrozole was released from the hydrogel within the first hour. Subsequently, the cumulative percentage of drug release exhibited a slight decrease with increasing anastrozole concentration. However, unlike BSA, the decrease in cumulative drug release was not as pronounced.

Again, the release kinetics of anastrozole was examined starting with zero-order kinetics by plotting the percentage of cumulative drug release against time. However, a linear relationship was not observed, indicating that the release profile does not adhere to zero-order kinetics (Figure 6). Next, we examined the Weibull model for anastrozole release by plotting Ln[−ln(1 − %Drug release)/100] vs. ln(t). However, no linear relationship was observed, indicating that anastrozole release from the 5% hydrogel did not follow the Weibull model. Additionally, we studied the kinetics of anastrozole release using the Higuchi model by plotting % cumulative anastrozole release vs. the square root of time. Again, no linear line was observed, suggesting that anastrozole release from the hydrogel did not follow the Higuchi model.

**Figure 7:**
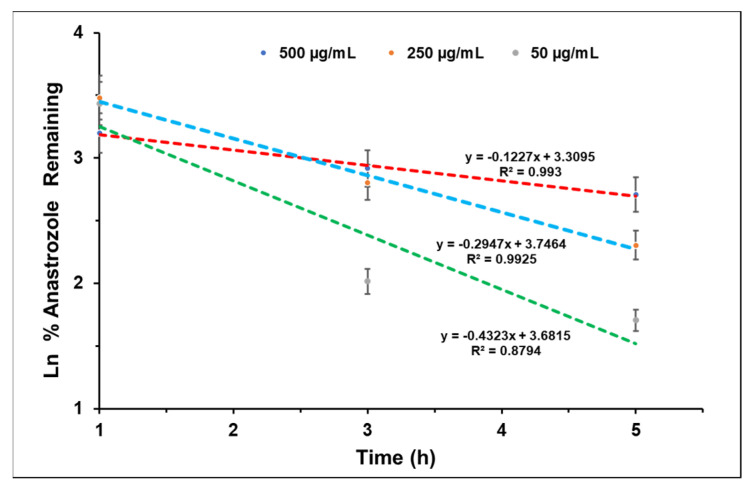
Kinetics of anastrozole drug release study from 5% hydrogel.

Finally, first-order kinetics were analyzed by plotting the natural logarithm of the percentage of remaining drugs against time, as illustrated in Figure 7. Notably, a linear plot was obtained, suggesting that the release of anastrozole follows first-order kinetics and is dependent on the concentration of anastrozole [59]. Thus, the release of anastrozole was similar to the release of BSA albeit at a faster rate.

The above in vitro loading and release of BSA and anastrozole study from the hydrogel shows that this hydrogel is useful for the release of both proteins and molecular drugs. By analyzing the kinetics of in vitro drug release study from hydrogel, the release of drugs occurs by diffusion and swelling controlled mechanism. In the diffusion-controlled mechanism, the concentrations of drugs are higher at the center of the hydrogel which allows for the diffusion or release of drugs initially at a high rate also called burst release and then slow release of drugs occurs from the pores or mesh of the hydrogel [60]. In the swelling-controlled mechanism, the polymers start to swell when the hydrogel is kept in PBS and start to degrade when it reaches the maximum extent thereby the burst and slow release of drugs occur [61]. In the case of the in vivo experiment, the drug should be injected with the hydrogel in a liquid state. The burst release of drugs may happen before settling down the gelling material, which gives immediate relief in the targeted area and then a slow release of drugs occurs [62].

We also tested the potential injectability of the hydrogel. The hydrogel still flows even after 2–3 min of mixing of two components to make the gel. Therefore, we believe it retains the injectability property for a reasonable duration. After mixing the solution, it takes approximately 5 min to form a stronger gel.

### 3.3. Biocompatibility Study

We conducted experiments to assess the potential of the hydrogel as a scaffold for promoting bone cell growth under in vitro conditions using mouse osteoblast MC3T3 cells as a model. These cells are widely used to test the biocompatibility of bone implant materials. In the case of bone defects, the regenerative capacity of bone can typically heal most defects. However, when the defect size exceeds the critical threshold, the healing process becomes impeded [63]. Autografts have been traditionally utilized for repairing bone defects of critical size in orthopedics because of their exceptional osteoconductivity, osteoinductivity, and osseointegration characteristics [64]. Nonetheless, autografts are associated with limitations such as inflammation, disease transmission, limited availability, and high costs [65]. To overcome these challenges, various scaffolds and polymeric materials have been explored [66]. Injectable hydrogels, in particular, have shown potential for tissue engineering of bone [67]. However, the application of injectable hydrogels for tissue engineering of bone is still in its early stages of development, which motivated us to develop a suitable material.

**Figure 8:**
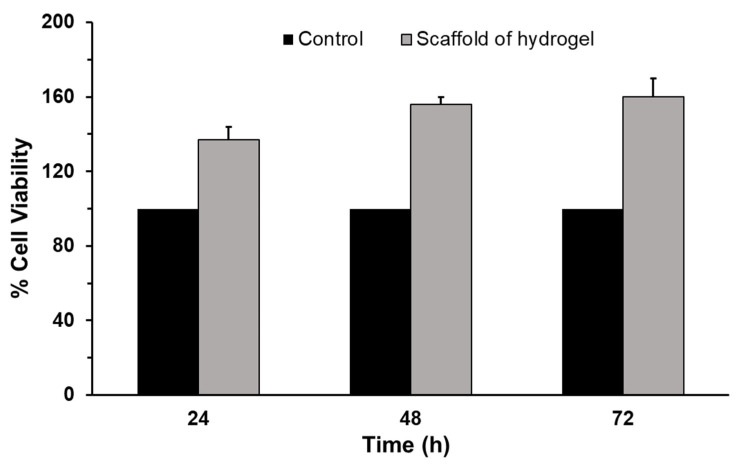
Osteoblastic cell (MC3T3-E1) growth on the control and scaffold of 5% hydrogel materials at different periods. Values shown are mean ± SD from at least three independent experiments each conducted in the quadruplicate sample. We used a one-way ANOVA test to evaluate statistical significance between experimental and control groups. The *p*-value of *p* ≤ 0.05 was taken as the value with a significant difference as compared with controls. Cell viability on scaffold hydrogel was significantly different from the controls at all time points tested (*p* ≤ 0.05).

To evaluate the performance of the hydrogel scaffold, mouse osteoblastic cell growth both on the scaffold of 5% hydrogel and a control substrate was studied (MEM). The cells were incubated at 37 °C for 24, 48, and 72 h (Figure 8). The results indicated that the cell viability percentage in the hydrogel scaffold after 24 h was 137, compared to 100 in the control group. Statistically, this value was significantly different compared to the control group. Furthermore, the cell viability rate increased significantly over time. After 72 h, the percentage of cell viability in the hydrogel scaffold reached 160, while it remained at 100 in the control group. These findings demonstrate the biocompatibility of the hydrogel and indicate that osteoblastic cells exhibit superior growth on the hydrogel scaffold compared to the control substrate.

**Figure 9:**
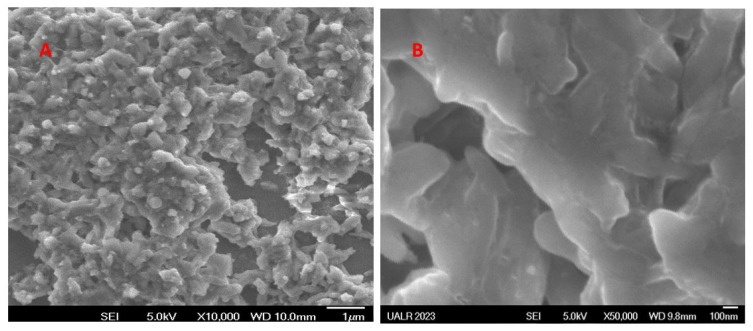
SEM images of the hydrogel scaffold. Hydrogel was spread over glass coverslips and dehydrated with increasing concentrations of ethanol. Shown in the figure are two representative SEM images at different magnifications showing porous structure of the hydrogel ((**A**). magnification 10,000×, (**B**). magnification 50,000×).

In order to determine whether the structure of the hydrogel scaffold was porous, scanning electron microscopy (SEM) analysis was performed. A thin layer of the hydrogel was spread over glass coverslips and analyzed by SEM following an ethanol dehydration protocol used to dehydrate MC3T3 cells gown on similar scaffold surfaces. SEM images (Figure 9A,B) taken at two different magnifications show that the surface of the scaffold hydrogel was porous indeed.

**Figure 10:**
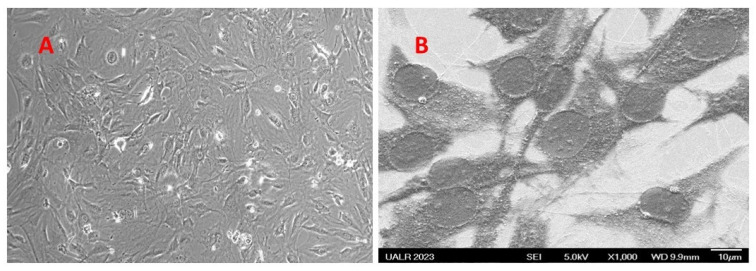
Morphological analysis of MC3T cells grown on hydrogel surface spread as a thin layer on glass coverslips. (**A**). shows the growth of the cells by light microscopy at 100× magnification. (**B**). shows the growth of the cells by SEM analysis at 1000×.

To study further whether the scaffold hydrogel surface supported bone cell growth, we studied the growth of the mouse osteoblastic MC3T3 cells on the hydrogel surface by light microscopy as well as SEM analysis. MC3T3 cells were grown on the hydrogel surfaces spread on the coverslips and placed in 6-well plates. For light microscopy, the cells were viewed under a light microscope after washing the cells with PBS, pH 7.4. The growth of the cells on the hydrogel surface is not expected to be seen by light microscopy because of the non-transparent nature of the hydrogel, which limits the passage of light through the gel. However, the growth of the cells over thin layers of the hydrogel was visible perhaps due to limited light passing through the translucent gel (Figure 10A).

For SEM analysis, the cells were fixed overnight with ice-cold methanol and then dehydrated with increasing concentrations of ethanol and time (40% -5 min, 50% -5 min, 60% -5 min, 70% -5 min, 80% -5 min, 90% -5 min, 95% -10 min and 100% -10 min) to remove any moisture. The coverslips were mounted on aluminum stubs using double-sided tape and made electronically conductive. The growth of the cells was then examined by scanning electron microscopy. As shown in Figure 10B, scaffold hydrogel supported bone cell growth. Cells grew evenly as monolayers over hydrogel surfaces and maintained the normal shape of MC3T3 cells. However, the granular and porous structure of the scaffold was not visible. Rather the surface looked smoother perhaps due to the filling up of the pores with cell culture materials and covering the granular surface by the cells. This supports biocompatibility and the utility of the hydrogel as a bone implant material.

## 4. Conclusions

The injectable hydrogel was synthesized through a host-guest interaction between p-βCD and Guar-ADI in a phosphate-buffered saline solution. Analytical techniques such as NMR, FTIR, and TGA confirmed the successful modification of the compound. Rheology analysis demonstrated the viscoelastic nature of the hydrogel. The release study of the hydrophilic (BSA) and hydrophobic (anastrozole) drugs from the hydrogel revealed a concentration-dependent decrease in cumulative drug release for BSA, indicating stronger interaction with the hydrogel, while anastrozole showed faster release due to weaker attachment to the hydrogel via hydrogen bond formation compared to BSA. Biocompatibility assessment with osteoblastic cells showed significantly enhanced cell viability compared to the control, with viability increasing over a 72 h period. These results highlight the hydrogel’s potential as an ECM material for tissue engineering of bone. Overall, this injectable hydrogel exhibits promising properties for controlled drug delivery and tissue engineering applications, offering opportunities for further development in the field.

## Data Availability

All data are kept in the corresponding laboratories and would be available upon request.

## Supplementary Materials

The following supporting information can be downloaded at: https://www.mdpi.com/article/10.3390/bioengineering10091088/s1, Figure S1: Fourier Transform Infrared (FTIR) spectroscopy of guar, 1-adamantyl isocyanate (ADI), and guar-ADI; Figure S2: Fourier Transform Infrared (FTIR) spectroscopy of β-cyclodextrin (β-CD), and poly-β-cyclodextrin (p-βCD); Figure S3: Proton Nuclear Magnetic Resonance (1H-NMR) spectroscopy of Guar-ADI; Figure S4: Proton Nuclear Magnetic Resonance (1H-NMR) spectroscopy of p-βCD; Figure S5: Carbon Nuclear Magnetic Resonance (13C-NMR) spectroscopy of Guar-ADI; Figure S6: X-ray Diffraction (XRD) of 5% hydrogel; Figure S7: Tan δ vs. angular frequency of 4%, and 5% hydrogels at room temperature; Figure S8: UV-Visible spectroscopy of 4% and 5% hydrogel; Figure S9: Scanning Electron Microscopy (SEM) (a. ×5000, b. ×100, and c. ×2000 magnifications) and d. Energy Dispersive Spectroscopy (EDS) of dried 5% hydrogel. Figure S10: Higuchi Model showing percentage (%) cumulative drug release vs. square root of time for BSA release study from 5% hydrogel having 1, 3, and 6 mg/mL BSA at 37 °C in PBS (7.4).
