# Supplementary material for: Guar-Based Injectable Hydrogel for Drug Delivery and In Vitro Bone Cell Growth
Source: Bioengineering (Basel). 2023 Sep 15;10(9):1088. doi: 10.3390/bioengineering10091088 (PMC10525255; doi:10.3390/bioengineering10091088)
Supplement: Supplementary file 1 [file bioengineering-10-01088-s001.zip › bioengineering-2589704-supplementary.pdf]

Article

Guar-Based Injectable Hydrogel for Drug Delivery and In Vitro Bone Cell Growth

Humendra Poudel [1], Ambar B. RanguMagar [2], Pooja Singh [3], Adeolu Oluremi [3], Nawab Ali [3], Fumiya Watanabe [4], Joseph Batta-Mpouma [5], Jin Woo Kim [5], Ahona Ghosh [1] and Anindya Ghosh [1,*]

[1] Department of Chemistry, University of Arkansas at Little Rock, 2801 South University Avenue, Little Rock, AR 72204, USA; hxpoudel@ualr.edu (H.P.); ahonag7@gmail.com (A.G.)
[2] Department of Chemistry, Philander Smith University, 900 W Daisy L Gatson Bates Dr, Little Rock, AR 72202, USA; arangumagar@philander.edu
[3] Department of Biology, University of Arkansas at Little Rock, 2801 South University Avenue, Little Rock, AR 72204, USA; pxsingh1@ualr.edu (P.S.); asoluremi@ualr.edu (A.O.); nali@ualr.edu (N.A.)
[4] Center for Integrative Nanotechnology Sciences, University of Arkansas at Little Rock, 2801 South University Avenue, Little Rock, AR 72204, USA; fxwatanabe@ualr.edu
[5] Department of Biological and Agricultural Engineering, Bell Engineering Center, University of Arkansas, 4183 Fayetteville, Little Rock, AR 72701, USA; jnbattam@uark.edu (J.B.-M.); jwkim@uark.edu (J.W.K.)
* Correspondence: axghosh@ualr.edu; Tel.: +501-916-5197; Fax: +501-569-8838

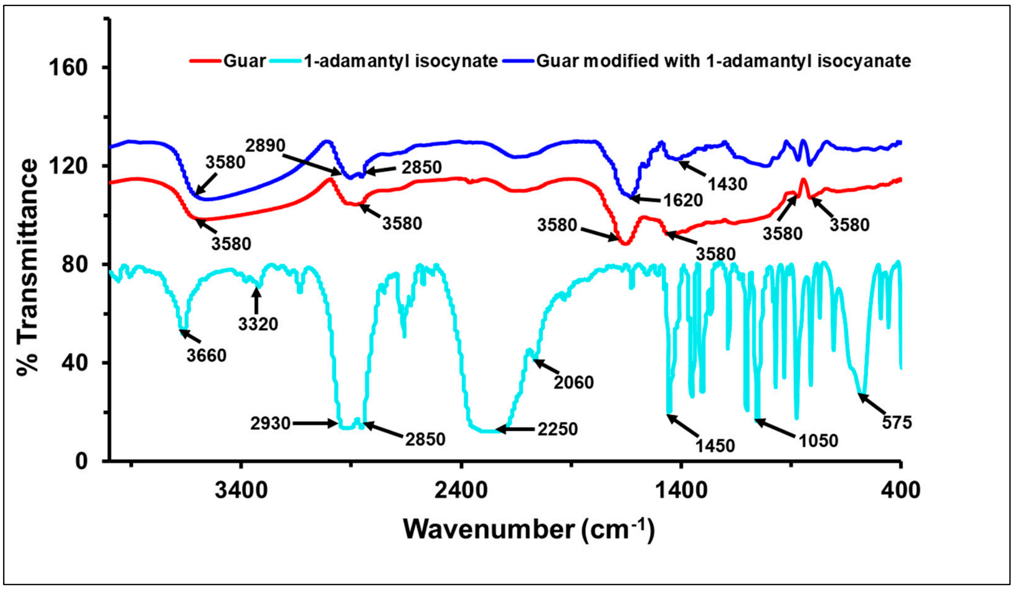

Figure S1. Fourier Transform Infrared (FTIR) spectroscopy of guar, 1-adamantyl isocyanate (ADI), and Guar-ADI.

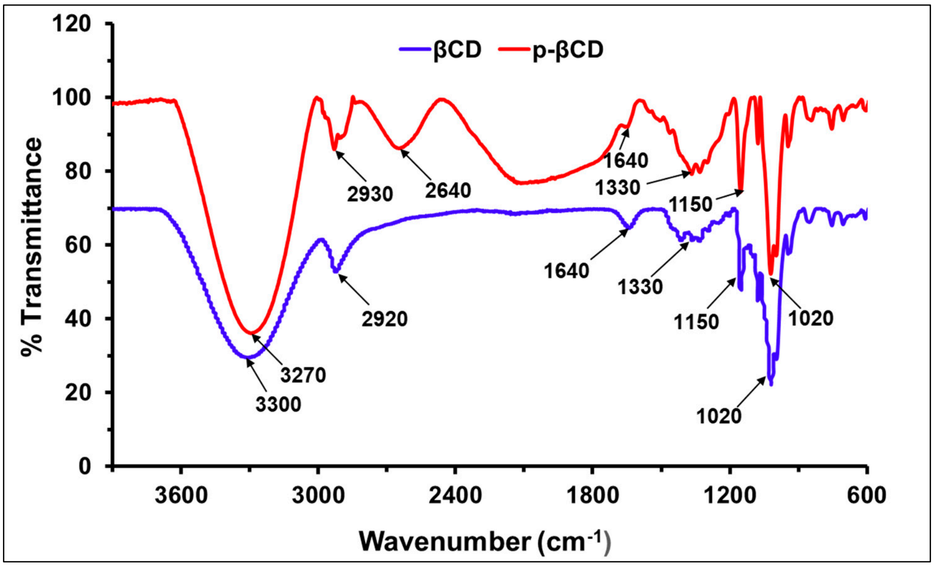

Figure S2. Fourier Transform Infrared (FTIR) spectroscopy of β-cyclodextrin (β-CD), and poly-β-cyclodextrin (p-βCD)

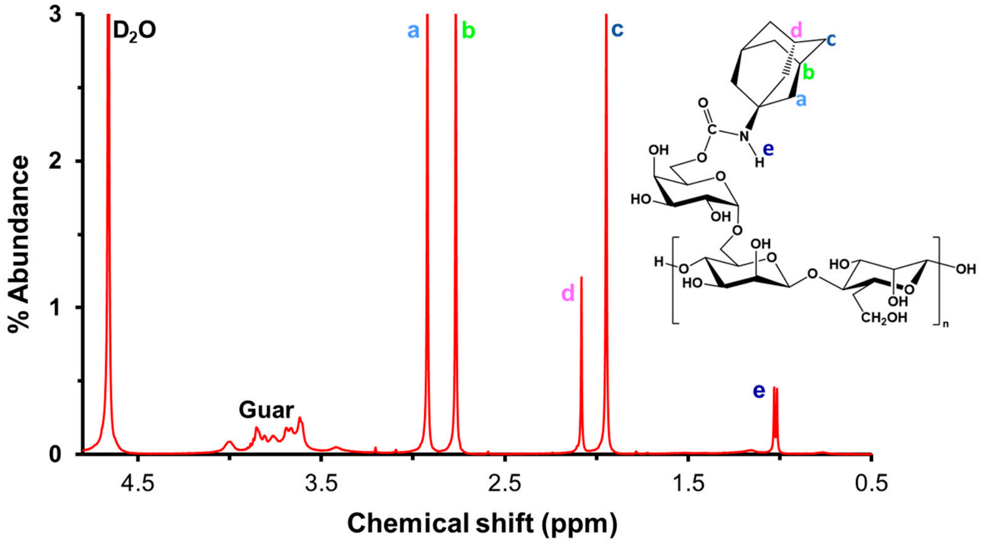

Figure S3. Proton Nuclear Magnetic Resonance (^{1}H-NMR) spectroscopy of Guar-ADI

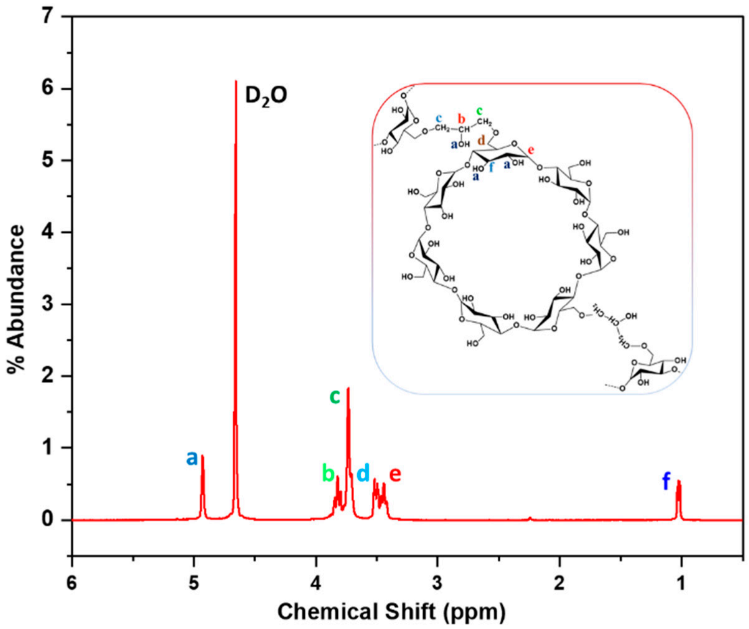

Figure S4. Proton Nuclear Magnetic Resonance (^{1}H-NMR) spectroscopy of p-βCD

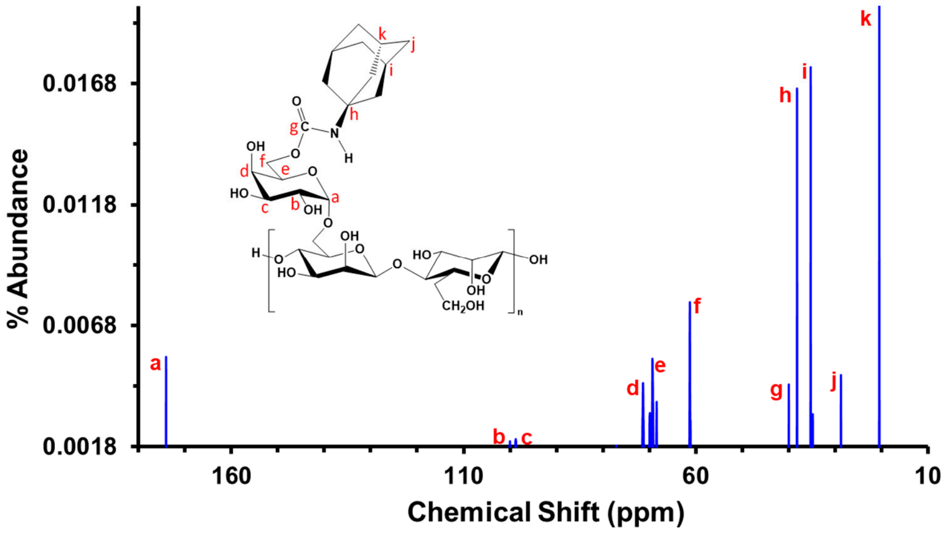

Figure S5. Carbon Nuclear Magnetic Resonance (^{13}C-NMR) spectroscopy of Guar-ADI

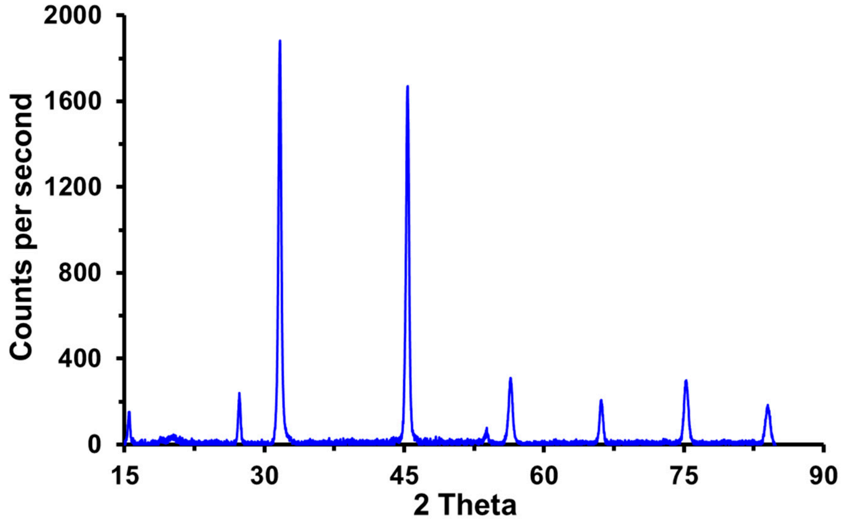

Figure S6. X-ray Diffraction (XRD) of 5% hydrogel

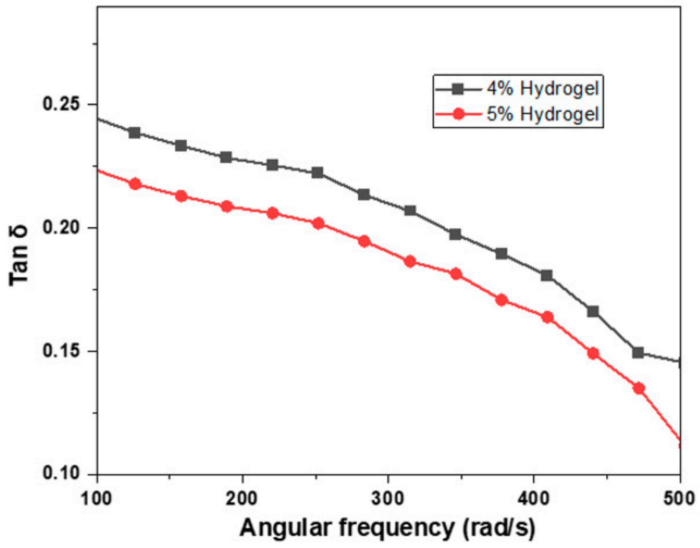

Figure S7. Tan δ vs. angular frequency of 4%, and 5% hydrogels at room temperature

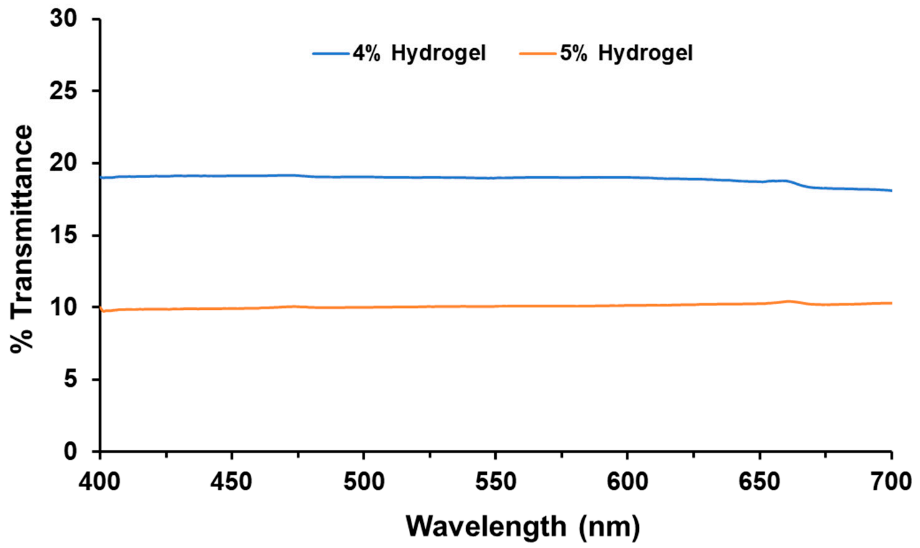

Figure S8: UV-Visible spectroscopy of 4% and 5% hydrogel

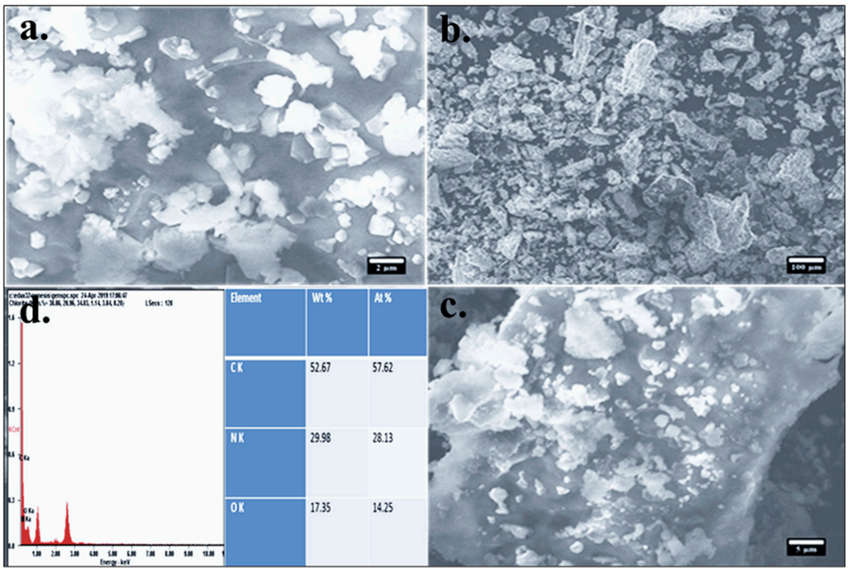

Figure S9. Scanning Electron Microscopy (SEM) (**a.** x 5000, **b.** x 100, and **c.** x 2000 magnifications) and **d.** Energy Dispersive Spectroscopy (EDS) of dried 5% hydrogel.

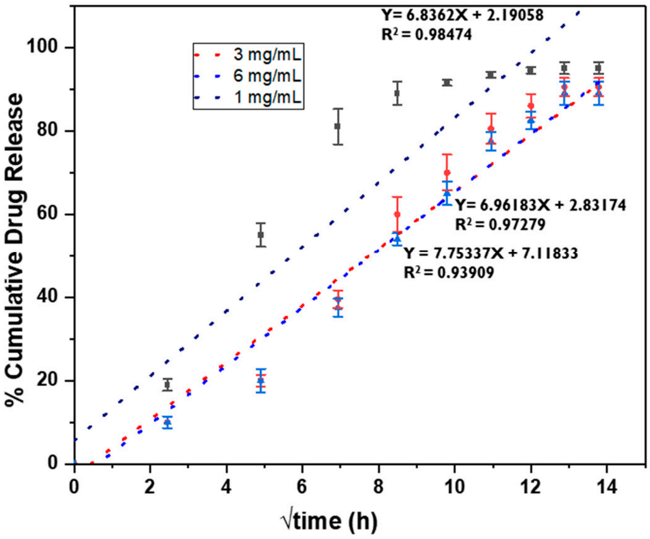

Figure S10: Higuchi Model showing percentage (%) cumulative drug release vs. square root of time for BSA release study from 5% hydrogel having 1, 3, and 6 mg/mL BSA at 37°C in PBS (7.4).
